# Remarks on Acute Rheumatism; with Cases

**Published:** 1827-02

**Authors:** Anthony Todd Thomson


### ACUTE RHEUMATISM.

*Remarks on Acute Rheumatism; with Cases.** By ANTHONY TODD THOMSON, M.D.

THE violence of the pain, the crusted state of the blood drawn from the arm, and the hard, quick, bounding, pulse which attend acute Rheumatism, have led medical writers to regard this disease as a purely inflammatory affection. Observation, however, and the experience of the present day, have justly thrown doubts upon the inflammatory nature of Rheumatism, and altered the mode of treating the disease. The use of the lancet is now rarely resorted to, except in cases where there is, naturally, a strong, rigid state of fibre, and that condition of the habit which is indicative of the inflammatory diathesis; for practitioners have found that bleeding in acute Rheumatism neither lessens the buffy appearance of the abstracted blood, however often the operation may have been repeated, nor diminishes the sufferings of the patient, unless the lancet be aided by narcotics, or other remedies which generally relieve pain.

The striking resemblance of acute Rheumatism to Ague was long since noticed by HULSE, MORTON, and many other eminent physicians, and treated with Cinchona bark on that account. The pain, when the disease is left to run its course undisturbed, recurs periodically every night or every morning, once at least in twenty-four hours. If the rigors be less obvious than in Ague, the sweats are frequently more so, and both are generally present in the rheumatic paroxysm. The appearance of the blood is the same in both diseases; and one of the most striking diagnostic marks in acute Rheumatism, the sediment deposited in the urine, is also present in Ague. The dread of metastasis led, we believe, to the discontinuance of the use of the bark as a remedy in acute Rhematism; but experience has demonstrated that this dread is unfounded, and where the employment of this remedy has been prefaced by the exhibition of Calomel, Tartar-emetic, and Opium, with cathartics, I have had no reason whatever, in a practice of twenty-five years, to alter my opinion of its efficacy and safety. The following cases, selected from my Case-book, show the manner in which I have exhibited it, and the result of its combination with other remedies.

*Case of Acute Rheumatism affecting the Forehead, (Cephalea auctorum.)*

November 20th, 1820.—Miss Francis G—, ætatis twenty-five, of a dark complexion, and tall, spare habit of body, with a profusion of black hair, had been for some weeks labouring under a slight catarrhal affection previous to the 12th instant, on the morning of which, after being exposed to a current of moist air on the previous evening, she suddenly felt a severe pain at the inner angle of the left orbit, directly behind the eyelid, which was slightly swelled. The pain gradually extended over the eyebrow, and along the brow: it continued until the evening, when it disappeared; but returned next morning, with as much severity as on the preceding day, and continued until the evening, when it again went off; and, in this manner, recurred daily up to the period of my advice being requested.

I found Miss G— suffering greatly from the pain; the eyelid was swelled; the eye itself slightly suffused; the pulse small, hard, and eighty; and the tongue covered with a white fur. The bowels were regular; the appetite not diminished; and, as she was free from pain, her nights were undisturbed. She was not sensible of any rigor or shivering at the accession of the pain, which always occurred before she rose from bed; but the paroxysm terminated with evident perspiration. The urine was scanty and high-coloured.

℞. Submuriatis Hydrarg. gr. viij.; Ext. Hyosciami gr. v. fiant pilulæ ij. horâ somni sumendæ.—℞. Sodæ Tartarizatæ ʒj.; Sodæ Carbonatis ʒss.; Infusi Sennæ f.ʒix.; Vini Colchici f.ʒj.; Tinct. Aurantii f.ʒj. M. ut fiat Haustus, in effervescentis actu c. Solutionis Acidi Tartarici cochl. ampl. j. primo cras mane et sextis horis sumendus.

21st.—The bowels have been actively purged, and much dark bilious matter passed. The pain, this morning, came on rather later than usual, but it is as severe as ever: both the eyelid and the forehead over the eyebrow are covered with a reddish tinge or blush; the eyeball is exquisitely painful, and the slightest pressure on the spot where the supraorbitary artery turns out of the orbit cannot be borne. The eye, also, of the affected side cannot be directed to the light, and is in a constant, involuntary, rolling motion. The tongue is more coated than yesterday; and the pulse eighty, small, and softer than yesterday. The temple throbs when the pain is severe.

℞. Submur. Hydrargyri gr. iv.; Antimonii Tart. gr. j.; Opii gr. vj.; Muc. Acaciæ q. s. ut fiant pilulæ æquales quatuor, quarum sum$^r$ j. sextis horis urgente dolore.—℞. Olei Terebinthinæ f.ʒiv.; Muc. Acaciæ f.ʒvj.; Pulv. Cinchonæ Cordifoliæ ℈ij.; Tinct. Cinchonæ comp. f.ʒiv.; Infusi Cinchonæ f.℥ivss.; Syr. Papaveris f.ʒij. M. ut fiat mistura, sum$^r$ cochl. amp. iij. quarta quâque horâ absente dolore.

22d.—She took three of the pills only, as the pain left her much earlier than usual. She does not nauseate the mixture, and has continued to take it up to this time, (ten o'clock A.M.) as the pain has not yet recurred. The bowels continue lax; the tongue is less furred; and the pulse only seventy-five, and fuller.

Pergat in usu medicamentorum.

23d.—The pain returned yesterday at three o'clock, but it was more supportable than before. She says she feels in every respect better. The oleum terebinthinæ has not affected either the kidneys or the skin, although the violet odour is present in the urine.

Contin[r] medicamenta.

24th.—Had no return of the pain yesterday, and considers that she is well. She was ordered to discontinue the pills, but to persevere in the use of the terebinthinate and bark mixture twice a-day, for four days; after which, there being no recurrence of pain, it was discontinued, and she was ordered to take, in its stead, the simple acidulated infusion of Cinchona for ten days. The disease did not return.

II. *Case of Acute Rheumatism affecting the whole Head.*

31st October, 1823, I was roused out of my bed to attend Miss C—, ætatis twenty-three, of a fair complexion, with light hair, a full habit of body, and endowed with great intellectual powers. The weather was damp, windy, and variable in temperature.

Miss C— complained of great pain over the whole of the head, so excruciating that she was afraid to go to sleep. This pain, which was preceded by a slight shivering, came on at ten o'clock this evening, and has continued to increase in violence until now (midnight). Her skin is not hot, her feet are as cold as marble; and she says that, besides the sensation of pain, her head feels as if it were screwed in a vice, and that it is sore to the touch. The pulse is small and slow; the tongue slightly furred; the bowels natural. The flow of the catamenia has scarcely ceased.

℞. Magnesiæ ʒss.; Vini Colchici m. xl.; Liquoris Anod. Hoffmani f.ʒj.; Misturæ Camphoræ f.ʒix. M. ut fiat Haustus quamprimum, et urgente capitis dolore, sumendus.—Let the pediluvium be used; and give a dose of Castor-oil in the morning.

1st November.—Two of the draughts were taken before the violence of the pain was abated; but Miss C— had no sleep, and the pain is now (ten o'clock A.M.) considerable. The Castor-oil has twice operated. The pulse is quicker and less languid than it was last night; the tongue is natural.

℞. Pulveris Cinchonæ Cordifoliæ ʒss. pulvis, ex Cyatho Aquæ, secundis horis sumendus.—The powders were ordered to be discontinued when the pain became very acute; and the draughts to be then repeated, as on the previous night.

2d.—The pain returned with great violence last night, although six of the powders had been taken. The pain is now more supportable; the pulse is small, and sixty; the bowels are open, and the urine deposits a copious pink sediment.

℞. Pulveris Ipecacuanhæ ʒss. pulvis emeticus accessione paroxysmo sumendus.—Cont[r] Pulveres, addendo sing. Pulv. Cinchonæ gr. x.

3d.—Miss C— had a very slight return of pain only after the operation of the emetic, and in the evening imagined that it had entirely disappeared; but it returned at midnight. The motions are pale and scanty. The tongue is cleaner; the pulse small, and sixty.

℞. Pilulæ Hydrarg. gr. xxiv.; Antimonii Tart. gr. jss.; Opii gr. vj. Contunde simul et divide massam in pil. quatuor, sum$^{r}$ j. omni nocte.—Contin$^{r}$ pulveres ut heri.

4th.—Had some sleep during the night, from the pill; but the pain returned at the usual hour this morning, although the paroxysm was much less violent and shorter than before.

Rep$^{r}$ pulv. u. a.—Cont$^{r}$ pilulæ.

5th.—There has been no pain to-day. The evacuations are more natural than usual.

Rep$^{r}$ pilulæ et pulveres u. a.

8th.—Miss C— thought that she felt the pain coming on this morning, but it was scarcely perceptible, and is now (twelve o'clock, noon,) completely gone. She appears in excellent spirits.

Cont$^{r}$ pulv. ter die.—Con$^{r}$ pil. anody. u. a.

13th.—The use of the bark powders has been continued since the 6th, and the pain has not returned. The patient is, indeed, quite well, both in bodily health and in spirits.

Let her discontinue the medicines, and commence the daily use of the shower-bath.

III. *Case of Acute Rheumatism accompanied with Cough.*

1st November, 1822.—Mrs. T—, ætatis twenty-six, of a spare habit of body, fair hair, and bilious temperament, had caught cold by sitting on the damp grass, to sketch a landscape from nature. She coughed often, but generally in paroxysms which recurred in the morning at 9 o'clock, and in the evening. She experienced some temporary relief from opiates, purgatives, and wine of Colchicum; but the cough had hung about her for upwards of two months, until two days since, when she was attacked with the most excruciating pain in the head and face, which left her short intervals only of ease, and returned periodically with greater violence. As the pain was extreme in the jaw, in the root of a carious tooth, this was extracted; but, although she felt some abatement of pain, and slept well for several hours after the operation, yet, on awaking, the pain was present. The throbbing in the site of the carious tooth was gone, but the general pain of the head was unabated. Her bowels had been previously well opened; the pulse was small, and there was not any preternatural heat of skin.

℞. Pulv. Cinchonæ Cordifoliæ ʒj.; Infusi Cinchonæ f. ℥ ij. M. fiat haustus tertia quâque horâ sumendus.

2d.— The pain is less violent, but it is not gone. The patient feels sick and languid this morning: the pulse is soft and feeble; the tongue is clean, and the bowels are regular.

Rep$^{r}$ pulveres u. a.

In the evening, the pain of the head returned, and she complained of sore-throat. On examining the fauces, the tonsils were seen to be slightly inflamed, but not enlarged.

℞. Tinct. Capsici f.ʒss.; Infusi Rosæ f. ℥ vj.; Mellis ʒiv.; Acidi Muriatici diluti m. xij. M. fiat gargarysma sæpe utendum.—℞. Submur. Hydrargyri gr. j.; Antimonii Tartar. gr. ¼; Opii gr. jss. fiat pilula horâ somni sumenda. —Let the feet be bathed in hot water.

I ought to have noticed that, whenever the head is easy, Mrs. T— is affected with cough, which leaves her as soon as the pain returns to the head.

3d November.—My patient is much freer from pain, but it still makes its attack twice in twenty-four hours. On abating, the pain leaves a sensation in the ear as if an imposthume was forming. The throat is free from inflammation; the pulse is sixty, and small; the urine deposits a copious pink sediment; the bowels are open.

Cont$^{r}$ pulveres et infusum u. a.

4th.—Mrs. T— had a very restless night. The tongue is dry, or rather has a dark brown streak in the middle of it, but the edges and about one-third of its breadth are moist. She complains of constant tenesmus. The pain of the head and the face is much less severe, and there is less throbbing in the ear.

℞. Magnesiæ ʒss.; Magnesiæ Sulphatis ʒiij.; Infusi Sennæ f.ʒxiij.; T. Rhei comp. f. ʒj. M. ut fiat haustus quamprimum sumendus.—℞. Pulv. Cinchonæ ℥ j.; Aquæ ferventis f. ℥ xij.; Acidi Sulph. d. f. ʒ ij. M. decoque per horam, in vase clauso, et cola.—℞. Liquoris Colati f.℥vss.; Tinct. Cinchonæ f. ʒ iv. M. fiat mist. cujus sum$^{r}$ tertiam partem quartis horis.

—The decoction, when strained, had no perceptible acid taste: thence I concluded that the acid combined with the Quinia, and formed a sulphate.

5th.—Nearly as yesterday; but some irritation is excited by spiculæ of the fractured bone of the socket of the tooth which was extracted. Mrs. T— still complains much of the ear.

Cont$^{r}$ mistura ut hieri.

6th.—Mrs. T— had a restless night, and is extremely nervous this morning. I have removed the spiculæ of the fractured jawbone, and ordered a fomentation of Chamomile-flowers and Poppy-heads to be applied over the ear and jaw. The bowels continue regular.

Rep$^{r}$ Mistura c. Cinchona u. a.—℞. Vini Colchici f.ʒj.; Liq. Anod. Hoff. f. ʒ ss.; Mist. Camphoræ f.ʒx.; Syr. Papaveris f.ʒss. M. fiat haustus horâ somni capiendus.

7th.—The pain, although not quite gone, yet is more supportable. The cough has not returned as usual, and is only troublesome on first going to bed.

Rep$^{r}$ medicamenta.

12th.—The use of the mixture has been continued up to the present time. The patient is in every respect better, the pain being scarcely felt, her rest sound, and the cough only troublesome in the morning.

20th.—Mrs. T— is now perfectly free from pain and cough, and considers that she is quite well.

IV. *Case of Acute Rheumatism, in which the influence of the Cinchona in removing the Symptoms was strikingly evident.*

1st November, 1822; the weather moist, and rather sultry for the season of the year; wind west by south-west.—Thomas Phillips, ætatis twenty-nine, a footman, of a spare habit of body, with black hair, and of a melancholic temperament. Being in the habit of perspiring freely at his work, he very imprudently sat down in a current of air to cool himself, a few days since, in the morning; and, in the evening of the same day, became affected with severe pain in his wrists and ankles. This pain, however, did not continue more than two days, and was succeeded by a very acute pain in the loins, and darting pains down the left leg. The pain, both in the loins and the leg, greatly increased at night; but, although it was much easier during the day, it never altogether left him. His skin is hot and dry in the evening, and he sweats so profusely at night that his shirt is quite wet, and he is obliged to rise and change it. The pulse is small and tense, and ninety; the tongue much furred in the middle, but clean and very red at the edges, and feeling as if it were scalded when he takes any thing warm into his mouth. The bowels were regular. Every day since the commencement of the disease, he has experienced a slight rigor about one o'clock in the afternoon, which is succeeded by heat of skin in the evening. He has taken two doses of salts.

℞. Submur. Hydrarg. gr. iv.; Micæ panis q. s. ut fiat pilula quamprimum sumenda.—℞. Magnesiæ ℈ij.; Vini Colchici f. ʒ j.; Infusi Sennæ f. ʒviij; Aquæ Anethi f.ʒvj. M. fiat haustus horâ post pilulam sumendus.—℞. Subm. Hydrarg. gr. j.; Antimonii Tart. gr. $\frac{1}{4}$; Opii gr. jss. Muc. Acaciæ q. s. fiat pilula h. s. sumenda.—℞. Olei Terebinthinæ f.ʒiij.; Muc. Acaciæ f.ʒv.; Pulv. Cinchonæ Cordifoliæ ℈ij.; Infusi Cinchonæ Cord. f.℥vss.; T. Cinchonæ comp. f.ʒiv.; Syr. Papaveris f.ʒij. M. ut fiat mist. cujus sum^r cochl. amp. iij. cras mane et quartis horis.

2d.—The bowels were freely purged by the Calomel and the purgative draught. He slept soundly at night, and awoke free from pain, but is very languid and feverish. His tongue, although clean at the sides, yet is covered in the middle with a dry, harsh, brown streak; it is also redder at the edges than it was yesterday. The pulse is slower, fuller, and softer than yesterday.

Cont^r pil. anod. et mistura.

3d.—Continues free from pain. He had a very restless night, but did not perspire. He has had one motion this morning. The urine is less high coloured, and is free from sediment. The tongue is much cleaner, moister, and less red on the edges. The pulse is soft, regular, and only sixty-eight. He forgot to take his pill last night.

Rep^r pilula u. a. et mistura sine Pulv. Cinchonæ.

4th.—He had a return of pain this morning, but in other respects remains as before.

Rep^r pil. et mist. addendo Pulv. Cinch. u. a.

5th.—Has lost the pain, and is in every respect much improved.

His urine is less high coloured, and deposits no sediment; the bowels are regular; pulse sixty-eight, but rather feeble; the tongue cleaner, but still too red.

Cont^r mistura.

6th.—Complains of nothing but debility. He begins to nauseate his medicine, which he says causes an unpleasant sensation of heat at the stomach, that continues for some time after it has been swallowed.

Let him leave off all medicine for twenty-four hours.

7th.—Had a very restless night, and perspired profusely. He remarked that he passed a great quantity of urine last night. To-day, he feels his head muddled and his mind confused. The tongue is more furred; the pulse small, soft, and sixty; the bowels are regular. He is quite free from pain.

℞. Infusi Cinchonæ f. ℥ vss.; Tinct. Cinchonæ comp. f. ʒ iv.; Acidi Muriatici diluti. m. xij. M. sum^r cochl. amp. iij. post sequentarum pilularum actionem et quartis horis.—℞. Subm. Hydrarg. gr. iv.; Ext. Colocynth. gr. ix. fiant pil. iij. quamprimum sumendæ.

12th.—Since the 7th, he has continued to improve daily, and is now in a state of convalescence. On account of the debility which remains, he takes two glasses of wine at noon.

I have selected these cases chiefly on account of the combinations in which the Cinchonæ was administered in them. The Oil of Turpentine, in particular, has always appeared to increase the efficacy of the Bark; and in very few instances, which have come under my notice, has it affected either the kidneys or the skin. In some habits, however, idiosyncrasy renders it impossible to continue the use of Turpentine longer than forty-eight hours. The urinary organs are strongly excited, and the urine is passed with great heat and pain, and occasionally tinged with blood; whilst the skin is covered with an eruption resembling Eczema rubrum: but, even in these individuals, the advantages derived from the remedy, in combination with Cinchona, counterbalances the inconveniences which it causes; although, assuredly, it should not be continued to be given when the eruption is fairly formed.

3, *Hinde-street, Manchester-square;*

18*th January*, 1827.

---

